# Cepharanthine and Curcumin inhibited mitochondrial apoptosis induced by PCV2

**DOI:** 10.1186/s12917-020-02568-0

**Published:** 2020-09-18

**Authors:** Yinlan Xu, Jiangang Zheng, Panpan Sun, Jianhua Guo, Xiaozhong Zheng, Yaogui Sun, Kuohai Fan, Wei Yin, Hongquan Li, Na Sun

**Affiliations:** 1 College of Veterinary Medicine, Shanxi Agricultural University, Taigu, Shanxi 030801 China; 2 Laboratory Animal Center, Shanxi Agricultural University, Taigu, 030801 Shanxi China; 3 Department of Veterinary Pathobiology, Schubot Exotic Bird Health Center, Texas A&M University, College Station, Texas, TX 77843 USA; 4 Medical Research Council (MRC) Centre for Inflammation Research, Queen’s Medical Research Institute, The University of Edinburgh, Edinburgh, EH16 4TJ UK

**Keywords:** PCV2, Cap, Paeonol, Cepharanthine, Curcumin, Apoptosis

## Abstract

**Background:**

Porcine circovirus type 2 (PCV2) is an immunosuppressive pathogen with high prevalence rate in pig farms. It has caused serious economic losses to the global pig industry. Due to the rapid mutation of PCV2 strain and co-infection of different genotypes, vaccination could not eradicate the infection of PCV2. It is necessary to screen and develop effective new compounds and explore their anti-apoptotic mechanism. The 13 natural compounds were purchased, with a clear plant origin, chemical structure and content and specific biological activities.

**Results:**

The maximum no-cytotoxic concentration (MNTC) and 50% cytotoxic concentration (CC_50_) of 13 tested compounds were obtained by the cytopathologic effect (CPE) assay and (3-(4,5-dimethyithiazol-2-yl)-2,5-diphenyltetrazolium bromide (MTT) method in PK-15 cells. The results of qPCR and Western blot showed that, compared with the PCV2 infected group, the expression of Cap in Paeonol (0.4 mg/mL and 0.2 mg/mL), Cepharanthine (0.003 mg/mL, 0.0015 mg/mL and 0.00075 mg/mL) and Curcumin (0.02 mg/mL, 0.001 mg/mL and 0.005 mg/mL) treated groups were significantly lowered in a dose-dependent manner. The results of Annexin V-FITC/PI, JC-1, Western blot and ROS analysis showed that the expression of cleaved caspase-3 and Bax were up-regulated Bcl-2 was down-regulated in Cepharanthine or Curcumin treated groups, while ROS and MMP value were decreased at different degrees and the apoptosis rate was reduced. In this study, Ribavirin was used as a positive control.

**Conclusions:**

Paeonol, Cepharanthine and Curcumin have significant antiviral effect. And the PCV2-induced Mitochondrial apoptosis was mainly remitted by Cepharanthine and Curcumin.

## Background

PCV2 is a DNA virus, which is an important pathogen causing high infection rate and immunosuppression in pigs [1]. The diseases caused by PCV2 mainly include postweaning multisystemic wasting syndrome (PMWS), Porcine dermatitis and nephropathy syndrome (PDNS), Porcine proliferative and necrotizing pneumonia (PNP), etc., which have caused serious economic losses to the global pig industry for about 57 years [2]. The capsid protein (Cap) encoded by the ORF2-encoded gene of PCV2 is a major viral structural protein and an immunogen involving viral replication of PCV2 [3]. Studies have shown that PCV2 could induce apoptosis and decrease spleen lymphocytes in immune organs of BALB/c mice and pigs [4, 5] and apoptosis can be induced through endoplasmic reticulum (ER) apoptosis, mitochondrial apoptosis, reactive oxygen species (ROS) regulation, etc. [6–9].

It has been reported that vaccination could not eradicate the infection of PCV2, due to the rapid mutation of PCV2 strain and co-infection of different genotypes, etc. [10, 11]. Therefore, it is needed to control infection. Chinese herbal medicine has the characteristics of high efficiency, low toxicity and low residue. It can improve the immunity, possess anti-stress and anti-virus ability. A variety of natural compounds or their active ingredients have been used to enhance host immune, prevent and treat viral diseases, such as Coronavirus diseases [12], Hepatitis B (HB) [13], Infectious bronchitis [14], African swine fever (ASF) [15], Influenza [16] and other viral diseases. However, the research on the anti-PCV2 action of natural compounds and its mechanism has also become a hotspot.

In our previous studies, dozens of natural compounds were screened for their antiviral activity, such as Chlorogenic Acid [17], ligustrazine hydrochloride [18], Scutellarin [17, 19], dipotassium glycyrrhizinate [18, 20, 21], Sodium tanshinone IIA sulfonate [21, 22], tea seed saponins [23] and Matrine [19, 24, 25]. Among them, only Scutellarin or Matrine had anti-PCV2 effect [19, 25].

Therefore, in this study, from many natural compounds with clear chemical structure, traditional medicine plant source and specific biological activity, 13 compounds with antiviral potential were selected and tested in the model of PCV2 infected PK-15 cell to screen anti-PCV2 compounds in vitro. Furthermore, the potential antiviral mechanism of compounds was explored and explained from the perspectives of apoptosis, mitochondrial membrane potential (MMP) and ROS. It provides a theoretical basis for the development of a safely, efficiently new compound with anti-PCV2 effect.

## Results

### Cytotoxicity of 13 compounds on the PK-15 cells detected by MTT

**Fig. 1:**
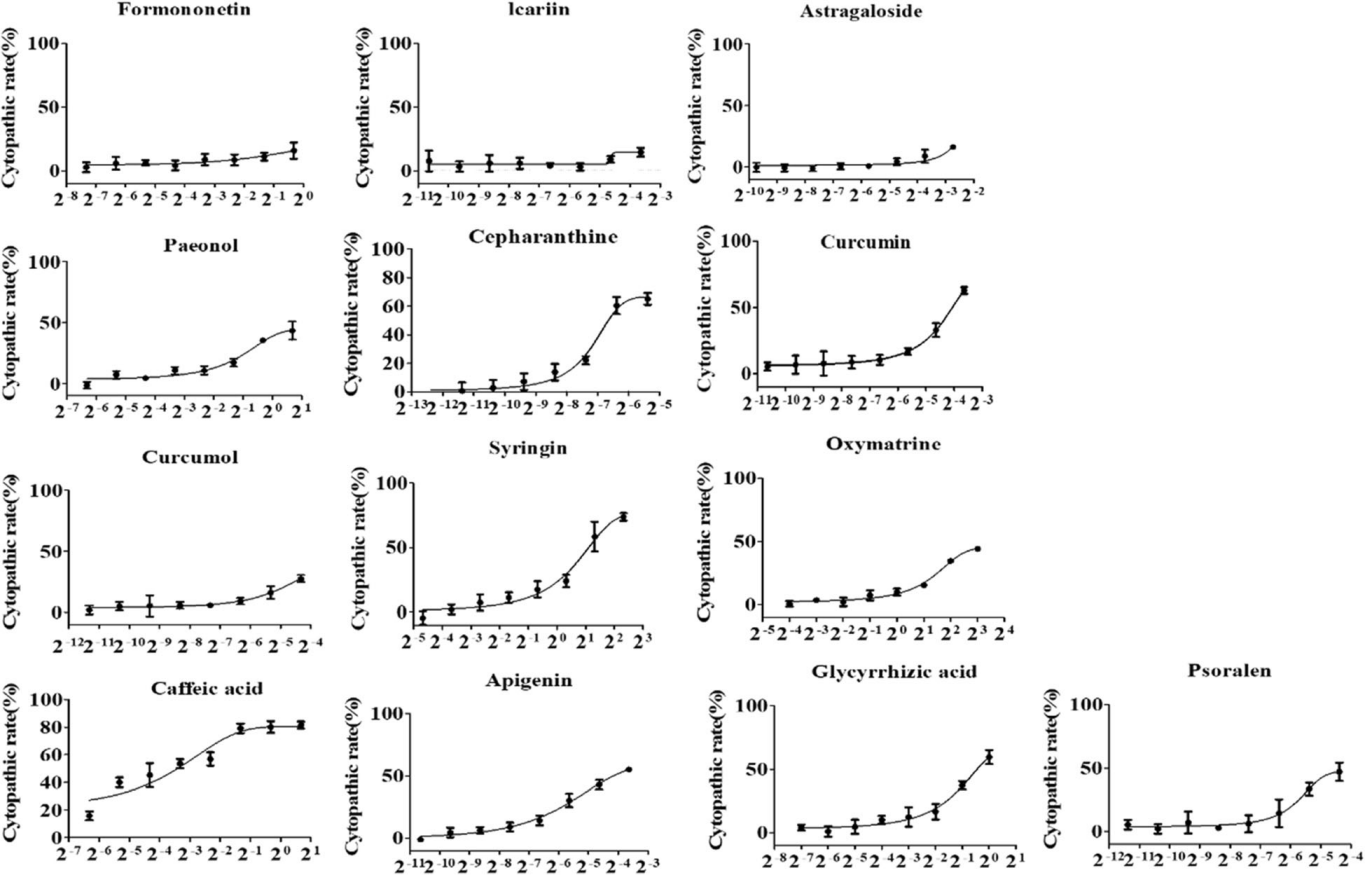
Dose-response curves of all tested compounds in cytotoxicity assay. 8 of gradient dilutions of each compound was prepared by 2-fold serial dilution and incubated with cells for 60 h. Dose-response curve of the tested compounds used in PK-15 shown the more cytopathic effect were observed with the increased concentration of compounds

Compared with the normal group of normal cells, the results showed that (See Additional file 1), the degree of cytopathological change were also different after the treatment of different compounds and different concentrations of the same compound. Due to the low solubility, the maximum solubility concentration (MSC) of 0.8 mg/mL Formononetin, 0.8 mg/mL Icariine and 0.15 mg/mL Astragaloside were used to treat the cells, respectively and no change in cell morphology was observed. The MNTC is the same as MSC. The 10 compounds except Formononetin, Icariine and Astragaloside showed different cytopathic changes (see Additional file 1) and MNTC values (Table 2). Dose-response curves of the tested compounds (Fig. 1) and CC_50_ value (Table 2) were generated by GraphPad Prism 5.0 analysis after calculating the CR value. The curves were “S” type, showing a dose-dependent relationship. As the concentration of the compound increased, more cells with change in morphology the was observed.

### The anti-PCV2 effect of Paeonol, Cepharanthine and Curcumin

**Fig. 2:**
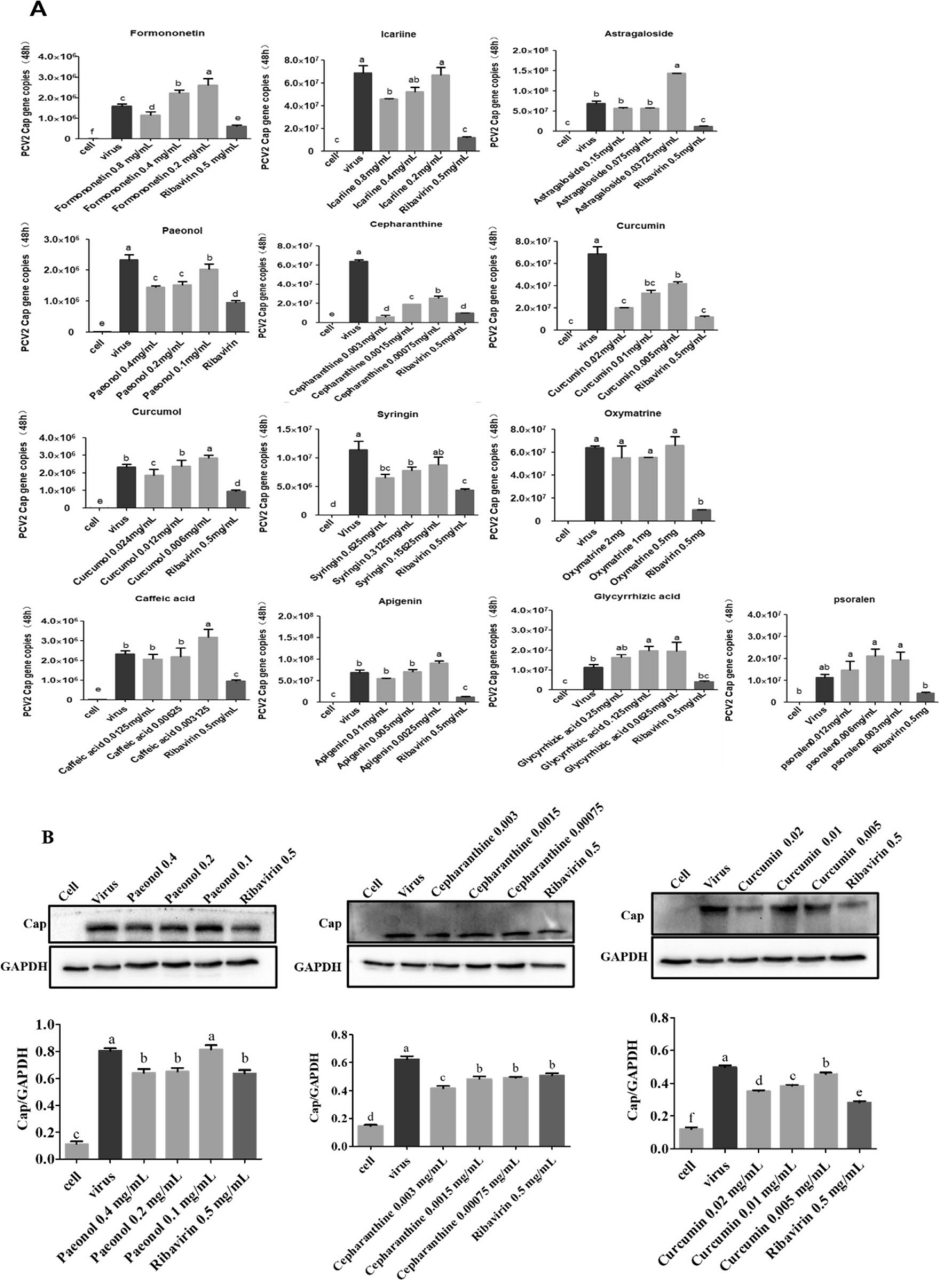
Anti-PCV2 activities of Paeonol, Cepharanthine or Curcumin detected by qPCR and Western blot. The anti-PCV2 effect of compounds was evaluated by qPCR and Western blot. Cells were incubated with 10^4.4^ TCID_50_ of PCV2 for 2 h, then tested compounds were added respectively and cultured for 48 h. Cells were collected and relative assay perform. **a** The expression of the *Cap* gene was detected by qPCR; **b** The expression of the Cap protein was detected by Western blot. GraphPad Prism 5.0 was used for the statistical analysis. All data were presented in Mean ± SEM. Different letters to indicate significant differences between groups (*p*<0.05)

To explore the anti-PCV2 effect of compounds, the expression levels of Cap was detected by qPCR and Western blot. The results of qPCR showed that, compared to cell control group, the PCV2 copy numbers were significantly increased (*P* < 0.05), compared to PCV2 infected group, the PCV2 copy numbers were significantly decreased in these compound treated with Formononetin at 0.8, Icariine at 0.8, Paeonol at 0.4, 0.2 and 0.1 mg/mL, Cepharanthine at 0.003, 0.0015 and 0.00075 mg/mL, Curcumin at 0.02, 0.01 and 0.005 mg/mL, Curcumol at 0.024 and Syringin at 0.625 and 0.3125 mg/mL (*P* < 0.05) (Fig. 2a).

Based on the results by qPCR, dose-dependent Paeonol, Cepharanthine and Curcumin were selected for Western blot. The results showed that the expression level of Cap protein treated with the three compounds respectively were significantly lower than PCV2 infected group (*P* < 0.05), except the 0.1 mg/mL of Paeonol group (Fig. 2b) (For original and unedited blots see Additional file 2). Therefore, the Cepharanthine and Curcumin were selected for the subsequent anti-apoptotic test.

### Inhibition of PCV2-induced apoptosis by Cepharanthine and Curcumin

**Fig. 3:**
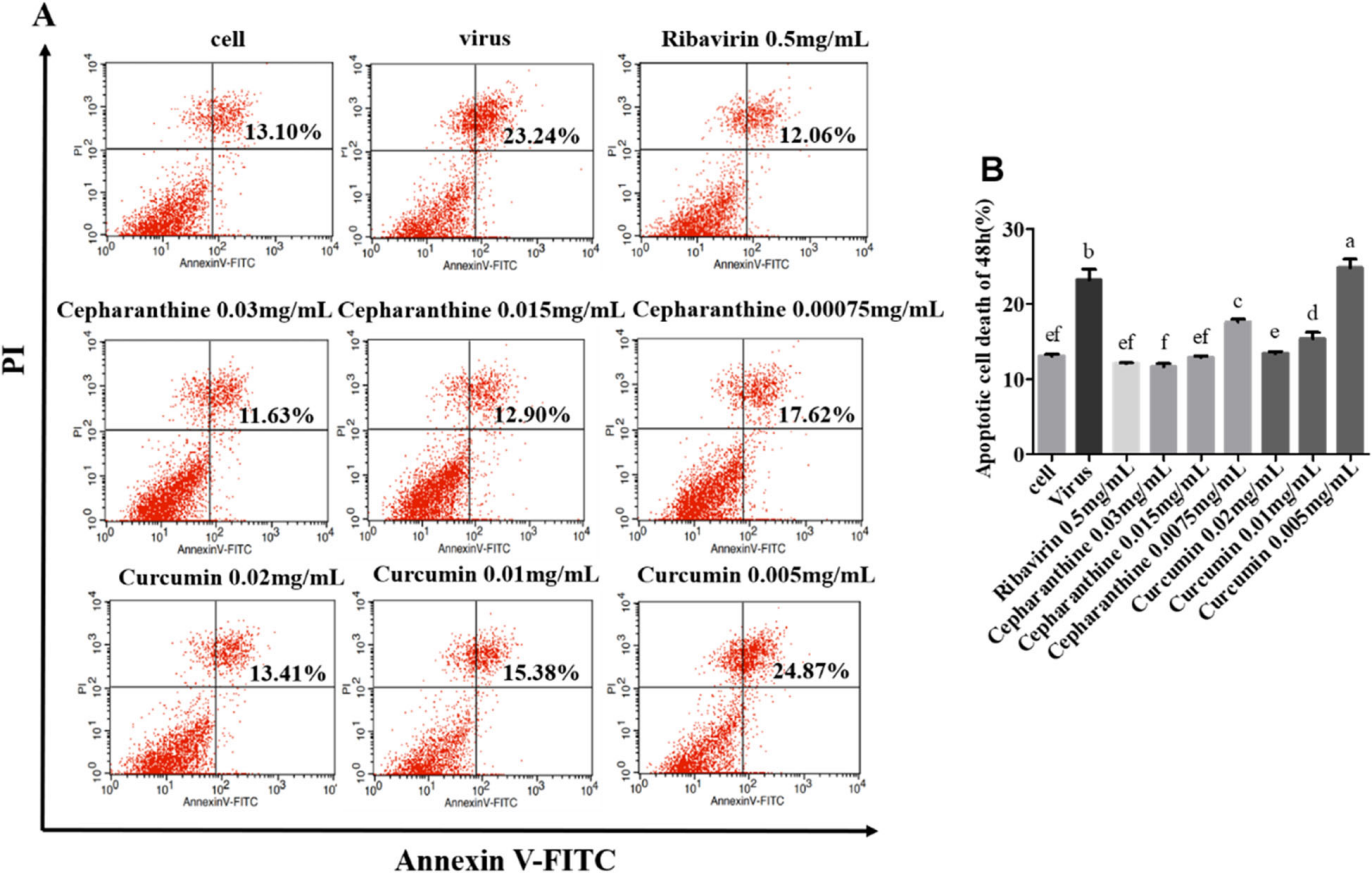
Inhibition of PCV2-induced cell apoptosis by Cepharanthine or Curcumin. **a** and **b** After cells were infected with 10^4.4^ TCID_50_ of PCV2 for 2 h, cells were treated with Cepharanthine, Curcumin or Ribavirin for 48 h. The apoptosis rate was evaluated by Annexin V/PI staining followed by flow cytometry. The data on the graph indicated the total number of cells at the late apoptosis of the right upper quadrant and the early apoptosis of the right lower quadrant. The results indicated apoptosis rates were significantly decreased in the treated group with Cepharanthine, Curcumin or Ribavirin Compared to the PCV2-infected group except the group of 0.005 mg/mL Curcumin (*P* < 0.05)

To evaluate the anti-apoptosis effect of Cepharanthine or Curcumin, Samples were analyzed after being treated with Annexin V/PI by Flow cytometer. The results showed that Compared to the PCV2-infected group, the cell apoptosis rates were significantly decreased in the group treated with Cepharanthine, Curcumin or Ribavirin, demonstrating a dose-dependent response except the group of 0.005 mg/mL Paeonol (*P* < 0.05) (Fig. 3a and b). Our results indicated that Cepharanthine, Curcumin or Ribavirin could significantly inhibit PCV2-induced apoptosis (*P* < 0.05).

### Inhibition of PCV2-induced apoptosis by Cepharanthine and Curcumin via the mitochondria pathway

**Fig. 4:**
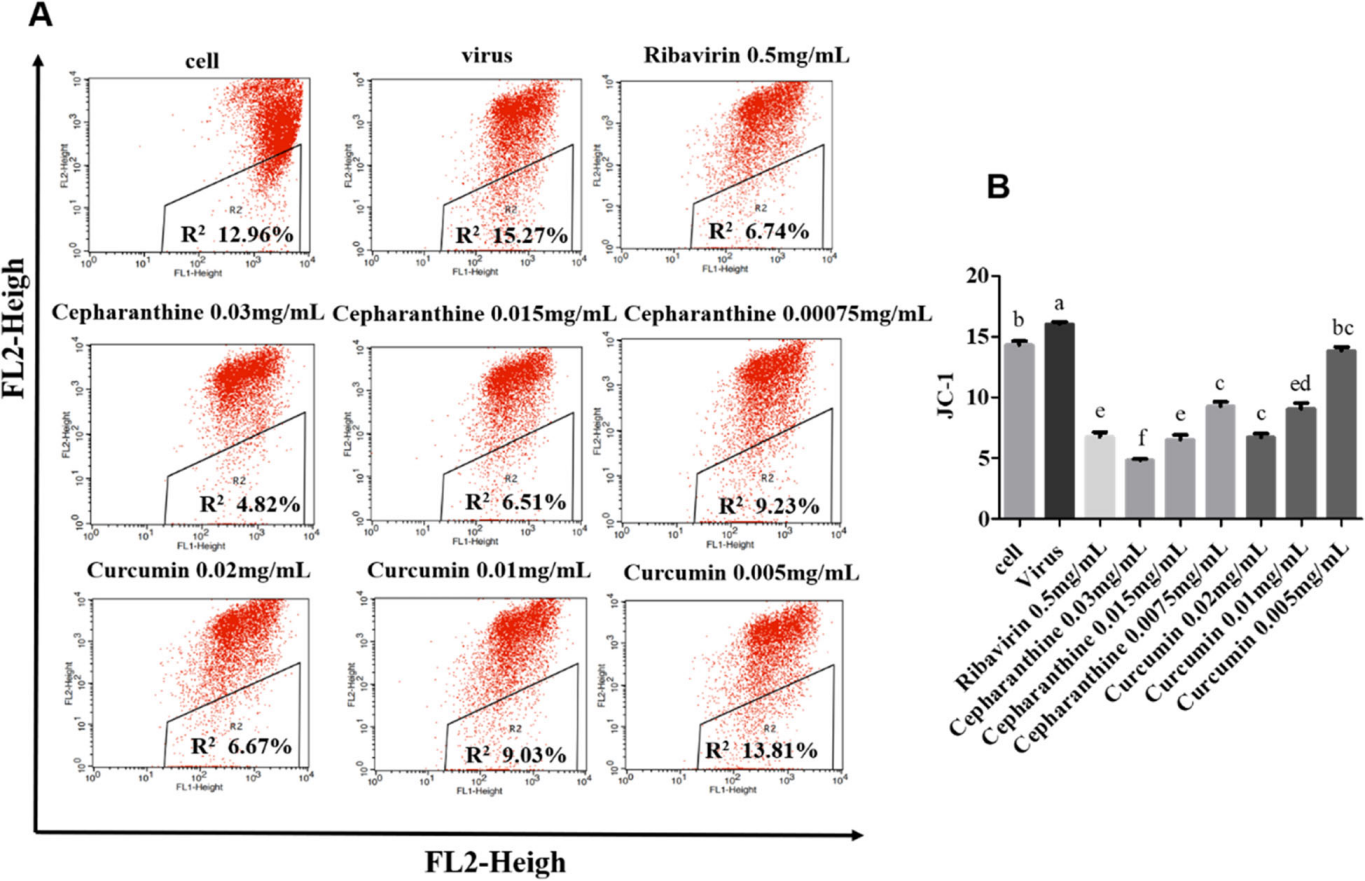
Inhibition of PCV2-induced cell apoptosis by Cepharanthine or Curcumin via the mitochondria pathway. **a** and **b** The changes of MMP in each group after being staining with JC-1 kit were detected by flow cytometry. R2 represents the value of MMP. The results indicated MMP values were significantly decreased in the treated group with Cepharanthine, Curcumin or Ribavirin Compared to the PCV2-infected group (*P* < 0.05)

To evaluate the anti-apoptosis mechanism of Cepharanthine or Curcumin, Samples were analyzed after being treated with JC-1 kit by Flow cytometer. The results showed that Compared to the PCV2-infected group, the MMP value were significantly decreased in the group treated with Cepharanthine, Curcumin and Ribavirin, demonstrating a dose-dependent response (*P* < 0.05) (Fig. 4a and b). The results indicated that Cepharanthine, Curcumin or Ribavirin could significantly inhibit PCV2-induced apoptosis via mitochondrial pathway (*P* < 0.05).

### The mechanism of inhibition of PCV2-induced mitochondrial apoptosis by Cepharanthine and Curcumin

**Fig. 5:**
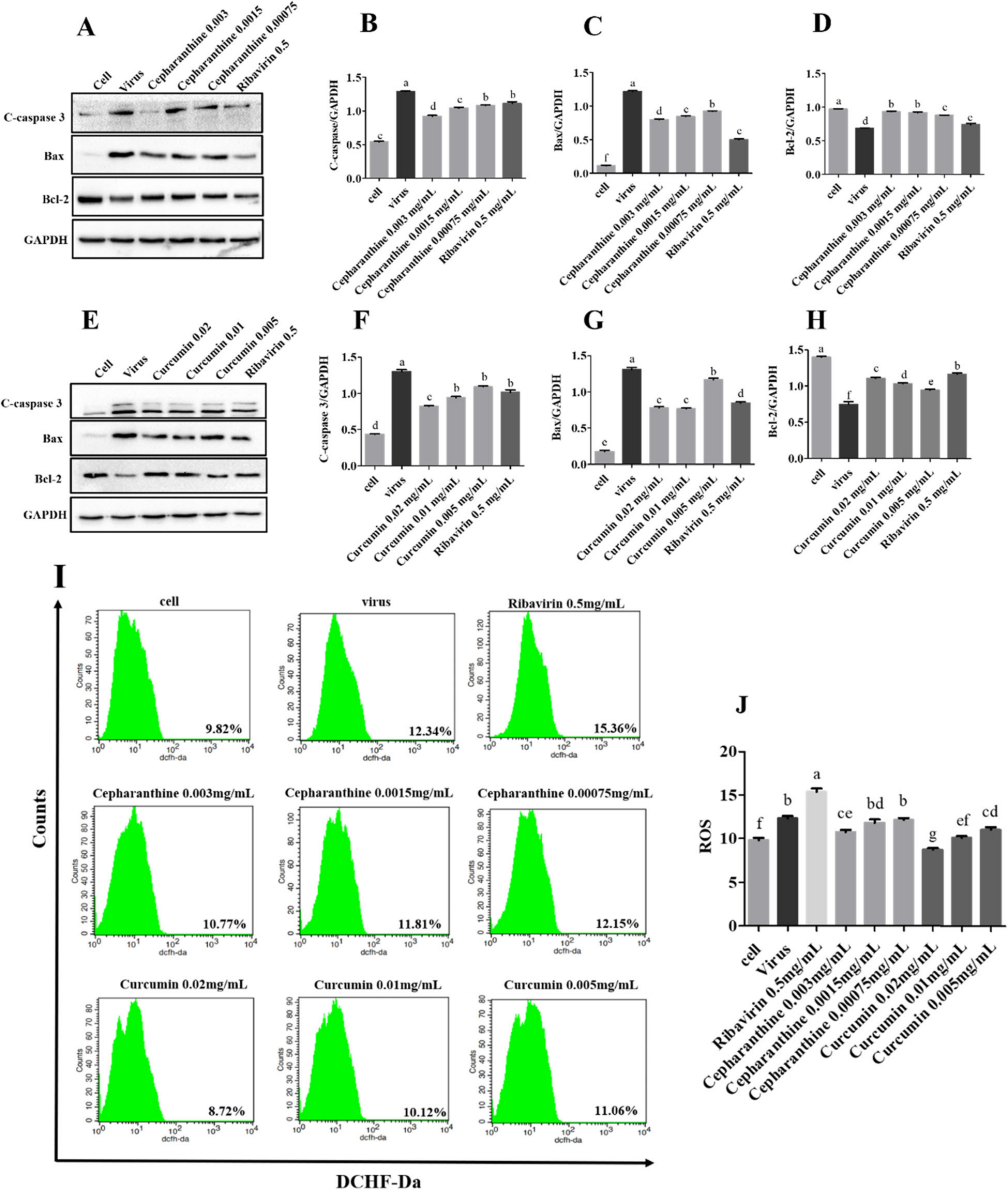
The mechanism of inhibition of PCV2-induced mitochondrial apoptosis by Cepharanthine or Curcumin. After cells were infected with 10^4.4^ TCID_50_ of PCV2 for 2 h, cells were treated with Cepharanthine, Curcumin or Ribavirin for 48 h. **a**-**h** The expression of the apoptin were analyzed by Western blot and grayscale analysis. **h**, **i** The results of flow cytometry showed that ROS values were significantly decreased in the treated group with Cepharanthine at 0.003 and Curcumin at 0.02, 0.01 and 0.005 mg/mL Compared to the PCV2-infected group (*P* < 0.05)

To evaluate the mechanism of inhibition of PCV2-induced mitochondrial apoptosis by Cepharanthine or Curcumin, Samples were analyzed by Western blot and Flow cytometer. The results of Western blot showed that Compared with the PCV2-infected group, the expression of pro-apoptin cleaved caspase-3 and Bax was significantly decreased while the expression of anti-apoptotic protein Bcl-2 was significantly increased in the treatment group of Cepharanthine, Curcumin or Ribavirin (Fig. 5a-h) (The original and unedited blots see Additional file 2). The results of Flow cytometer after being stained with ROS kit demonstrated that the value of ROS in the Curcumin-treated group showed strongly downward trend in a dose-dependent manner, compared with the PCV2 control group (Fig. 5i and j). However, only the group of treated with 0.003 mg/mL of Cepharanthine could significantly reduce the amount of ROS (*P* < 0.05). The results demonstrated that Cepharanthine or Curcumin inhibited the PCV2-induced increase in MMP through the caspase family and Bcl-2 family mechanisms. Curcumin inhibited PCV2-induced mitochondrial apoptosis by reducing ROS, However, ROS reduction is not the main pathway for Cepharanthine and Ribavrin to inhibit PCV2-induced mitochondrial apoptosis.

## Discussion

PCV2 is the main pathogen causing porcine circovirus associated diseases (PCVAD). In this study, 13 natural compounds with defined chemical structures, useful clinical effect and from medicinal plants were selected. After the primary screening, three compounds with antiviral effect, Paeonol, Cepharanthine and Curcumin were further studied. The anti-apoptotic mechanism of these two compounds was investigated by evaluating the level of key apoptins, MMP and ROS in the cellular mitochondrial pathway. Ribavirin has a broad antiviral activity and was used as a positive control.

Cepharanthine is a natural alkaloid extracted from the roots of the Stephania japonica, which has anti-inflammatory [26], anti-Hepatitis B virus [27], herpes simplex type-1 virus (HSV-1) [28] and etc. Curcumin is an active ingredient extracted from the rhizome of some plants of the Zingiberaceae and Araceae. Medical research shows that curcumin has low toxicity, little adverse reactions and multiple therapeutic effects, such as anti-inflammatory [29], anti- oxidative [30], anti-H9N2 influenza virus [31], dengue virus [32], hepatitis C virus [33] and so on.

Zhang et al. demonstrated that PCV2 Cap could induce mitochondrial apoptosis of PK-15 and PAM cells by interfering with Ca^2+^ homeostasis, down-regulating MMP and increasing intracellular ROS [7]; Lin et al. found that PCV2 induced mitochondrial apoptosis of PCV2 infected PK-15 cells and 293 T cells by increasing the activities of caspase-3 and caspase-9 and accelerating the release of cytochrome C (Cyto C) from mitochondria to cytoplasm [34]; After PCV2 infected RAW264.7 cells, stress-related molecules such as ROS production and NO secretion were significantly increased [35]; Previous studies have shown that PCV2 infection promotes the accumulation of ROS, which in turn inhibits the replication of PCV2 through the NF-κB pathway [36]. Therefore, we hypothesized that the experimental compounds could inhibit the mitochondrial apoptosis of PK-15 cells induced by PCV2 infection via acting on mitochondrial membrane potential. Mitochondria have a central role in cell apoptosis. The loss of MMP is the marker of mitochondrial apoptosis, the changes of MMP are caused by Bcl-2 family, Caspase family and Miochondrial permeability transition pore (MPTP) [37]. Cleaved caspase-3 is the main executive protein of apoptosis. There are many members of the Bcl-2 family, among which Bax is the main pro-apoptotic protein and Bcl-2 is the main anti-apoptotic protein. The opening and closing of MPTP is influenced by ROS, Ca^2+^, ADP, oxidative stress, high PH value and other apoptotic factors [37]. In this study, at first, the cell apoptosis and change of MMP were detected by flow cytometry after Annexin V/PI double staining and JC-1 assay. The results indicated that Cepharanthine, Curcumin or Ribavirin could significantly inhibit PCV2-induced apoptosis via mitochondrial pathway. To further evaluate the mechanism of Cepharanthine or Curcumin inhibiting mitochondrial apoptosis, the apoptins of Caspase family and Bcl-2 family were analyzed by Western blot, the values of ROS were analyzed by flow cytometry after ROS kit staining. The results showed that Compared with cell control group, the expression levels of cleaved caspase-3 and Bax were up-regulated, and the expression of Bcl-2 was down-regulated, the ROS values are increased. This result is consistent with the previous reports [6, 34, 38]. Compared with PCV2-infected group, the expression levels of cleaved caspase-3 and Bax were down-regulated, and the expression of Bcl-2 was up-regulated in the treated with Cepharanthine or Curcumin. The value of ROS was decreased but ROS did not decrease in treated with the 0.0015 and 0.00075 mg/mL of Cepharanthine. The results demonstrated that Cepharanthine or Curcumin inhibited the PCV2-induced increase in MMP through the caspase family and Bcl-2 family mechanisms. Curcumin could inhibit cell mitochondrial apoptosis by regulating the opening of MPTP through reducing the level of ROS, but Cepharanthine and Ribavirin did not have the similar effect. It is speculated that various antiviral compounds could inhibit the cell apoptosis by governing MMP through affecting mitochondrial apoptosis pathway.

## Conclusion

**Fig. 6:**
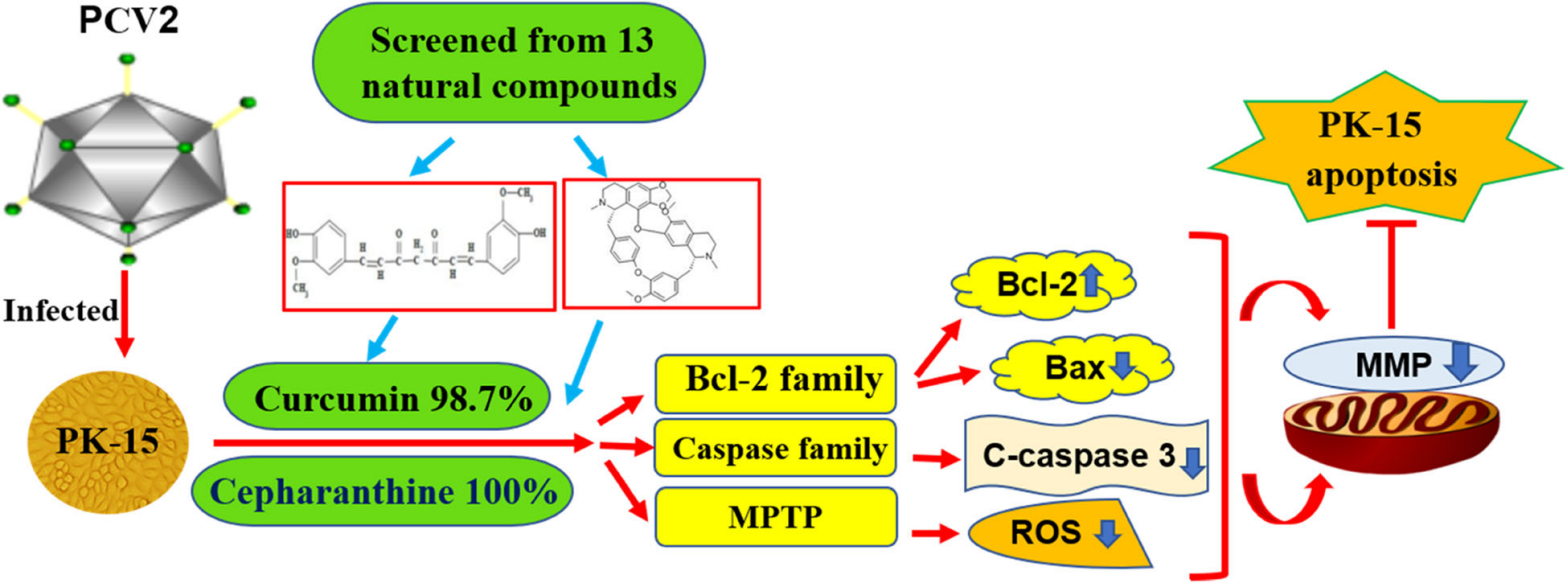
The PCV2-induced Mitochondrial apoptosis was mainly remitted by Cepharanthine and Curcumin. The “red arrow” represents the promoting effect and the red “T-shape” indicates the inhibiting effect. The PCV2 replication is inhibited by Cepharanthine and Curcumin, selected from 13 natural compounds, through mitochondrial apoptosis pathway

To summarize, our study revealed that Paeonol, Cepharanthine and Curcumin have significant antiviral effect, and Cepharanthine and Curcumin could inhibit PCV2-induced mitochondrial apoptosis by through change the MMP (Fig. 6). The study provides a base line for further studies on the anti-PCV2 effect and anti-apoptosis mechanism of Cepharanthine and Curcumin in experimental animal models infected with PCV2, and may be applied in clinical treatment as anti-virus compounds eventually.

## Methods

### Cells, viruses, antiboties and nature compounds

PCV-free pig kidney endothelial cell line (PK-15) was preserved in our laboratory. Cells were cultured and passed in Dulbecco’s modified eagle’s medium (DMEM, Hyclone, USA) containing 10% fetal calf serum (FBS, BI, Israel) (10% DMEM) and maintenance in DMEM containing 2% FBS (2% DMEM).

The strain of PCV2-SH (GenBank: AY686763.1) was gifted by Professor Jiang Ping of Nanjing Agricultural University. Virus was replicated and harvest in PK-15 cells infected with PCV2. The titer of 10^6.4^ TCID_50_/ mL was determined by Indirect Immunofluorescence (IFA).

Antibodies against Cap were purchased from Biorbyt LLC. (California, Britain) cleaved caspase-3, Bcl-2 and Bax were purchased from Abcam (Cambridge, USA); GAPDH and horseradish peroxidase (HRP)-conjugated secondary antibodies were purchased from Wuhan Sanying Biology Technology Co., Ltd. (Wu Han, China).

**Fig. 7:**
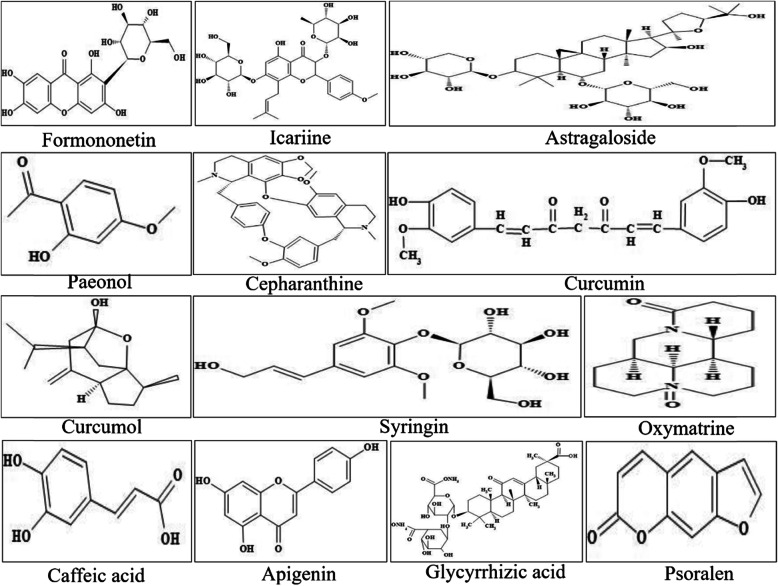
The chemical structure of 13 natural compounds

Ribavirin, the positive control drug, was purchased from Beijing Solarbio technology co., LTD; Syringin was purchased from Nanjing Puyi biotechnology co., LTD; Glycyrrhizin aid was purchased from DOSF biotechnology co., LTD; Other tested compounds, such as Curcumol, Paeonol, Oxymatrine, Caffeic acid, Formononetin, Cepharanthine, Apigenin, Psoralen, Lcariine, Curcumin and Astragaloside were purchased from National Institutes for Food and Drug Control. Compounds information and chemical structure were respectively listed Table 1 and Fig. 7.

**Table 1:** The compounds used in the test

No.	Chemical name	Classification	Main plant source	Cosolvent	Purity
1	Formononetin	Phenolic acids	Mango	1%DMSO	98.10%
2	Icariine	Flavonoid glycosides	Herba epimedii	1%DMSO	94.20%
3	Astragaloside	Saponins	Astragalus membranaceus	1%DMSO	96.90%
4	Paeonol	Monoterpenes	Peony	1%DMSO	99.90%
5	Cepharanthine	Alkaloide	Stephania	1%DMSO	100.00%
6	Curcumin	Polyphenols	Turmeric	1%DMSO	98.70%
7	Curcumol	Hemiketal	Curcuma zedoary	1%DMSO	99.90%
8	Syringin	Phenylpropanolides	Lilac	DMEM	95.20%
9	Oxymatrine	Alkaloid	Sophora	DMEM	92.50%
10	Caffeic acid	Organic acid	Caffeic	1%DMSO	99.70%
11	Apigenin	Flavonoid	*Apium graveolens*	1%DMSO	99.20%
12	Glycyrrhizic acid	Triterpenes	Glycyrrhiza	DMEM	95.00%
13	Psoralen	Furancoumarins	Fructus psoraleae	1%DMSO	99.70%

### Cytotoxicity assay

The viability of PK-15 cell was assessed with MTT. 1.0 × 10^6^ cells/mL of cells were seeded into 96-well plates and incubated until 100% confluency was reached. The tested compounds were added with two-fold serial diluted and cultured for another 60 h and cell control group was established. Four repeated wells were set up for each concentration with 100 μL per well. The cytopathic condition of cells was observed every day and photographed. The compounds and dissolution methods are shown in Table 1. Then 25 μL MTT (4 mg/mL in PBS) was added to each well and incubated at 37 °C for 4 h. 150 μL of DMSO (Solarbio, Beijing) was added and incubated for 30 min after removing the culture medium. The optical density (OD) were measured at 490 nm using a microplate spectrophotometer (Spectra Max M5, Molecular Devices, USA). Cytopathic ratio (CR) was calculated based on CR = [(OD control - OD test)/ OD] × 100. Then MNTC and CC_50_ on PK-15 cells were calculated using GraphPad Prism™ 5.0 (Inc. California, USA).

### Real time quantitative PCR

The anti-PCV2 effect of compounds in PK-15 cell was determined by real time quantitative PCR (qPCR). When cell confluency rate of 24-well plate reached 80%, 10^4.4^ TCID_50_ of PCV2 was incubated for 2 h and discarded the supernatant. MNTC of every test compound was in two-fold serial dilution with 2% DMEM into 3 gradients and added to 24-well plates (500 μL/well) (Table 2). Meanwhile, the cell control group, PCV2-infected group and Ribavirin positive control group were applied and incubated for 48 h with 5% CO_2_ at 37 °C. DNA was extracted according to the instruction of DNA extraction kit (TianGen, Beijing, China) and the DNA concentration was measured using a nucleic acid concentration analyzer (NanoDrop Technologies, Wilmington, DE, USA). The copy number of the *Cap* gene was detected by qPCR. The Primers 5′ TAC ATT TCC AGC AGT TTG and 5’CTC CCG CCA TAC CAT AA were used for the amplification of 148 bp fragment.

**Table 2:** The results of cytotoxicity

No.	Chemical name	MNTC(mg/mL)	CC_50_(mg/mL)
1	Formononetin	0.8	>0.8
2	Icariine	0.8	>0.8
3	Astragaloside	0.15	>0.15
4	Paeonol	0.4	1.2696 ± 0.08983
5	Cepharanthine	0.003	0.008048 ± 0.0006136
6	Curcumin	0.02	0.06296 ± 0.02424
7	Curcumol	0.024	0.115 ± 0.02331
8	Syringin	0.625	1.128 ± 0.5339
9	Oxymatrine	2	8.31 ± 0.3896
10	Caffeic acid	0.0125	0.2581 ± 0.07863
11	Apigenin	0.01	0.04817 ± 0.01821
12	Glycyrrhizic acid	0.25	0.7162 ± 0.04979
13	Psoralen	0.012	0.05549 ± 0.01850

### Western blot

The anti-PCV2 effect and anti-apoptotic mechanism of the compound was detected by Western blot. When cell confluency of 6-well plate reached 80%, 10^4.4^ TCID_50_ of PCV2 was incubated for 2 h and discarded the supernatant. Paeonol, Cepharaanthine and Curcumin were added in turn, 2 mL/well. The cell control group, PCV2-infected group and Ribavirin positive control group were applied and incubated for 48 h. Total cell protein was extracted and protein concentration was determined using the BCA protein assay kit (Beyotime Biotechnology, Jiangsu, China). The protein samples were separated by SDS-PAGE, then transferred to the PVDF membrane. The levels of PCV2 Cap protein and apoptin were detected using an eECL Western Blot detection kit (Cwbio Inc., Beijing, China) and chemiluminescence imaging system (BIO-RAD, California, USA).

### Annexin V/PI staining for apoptosis

The anti-apoptotic effect of Cepharaanthine and Curcumin were detected by Annexin V/PI. Cells in 6-well plate were incubated with 10^4.4^ TCID_50_ of PCV2 for 2 h and discarded the supernatant, then Cepharaanthine and Curcumin were added, the cell control group, PCV2-infected group and Ribavirin positive control group were applied and incubated for 48 h. Samples were collected, centrifugated and treated using Annexin V/PI kit (Keygen Biotech, Nanjing, China), then analyzed by flow cytometry (BD Biosciences, USA).

### Detection of MMP by JC-1

Mitochondrial membrane potential (MMP) of cells was detected by JC-1. Cells in the 6-well plate were incubated with 10^4.4^ TCID_50_ of PCV2 for 2 h and discarded the supernatant, then Cepharaanthine and Curcumin were added. After 48 h incubation, cells were stained with JC-1 using a commercial kit (Beyotime Biotechnology, Jangsu, China) treated and analyzed by flow cytometry (BD Biosciences, USA).

### Detection of ROS by flow cytometer assay

The amount of reactive oxygen species (ROS) was measured by flow cytometry. Cells in the 6-well plate were incubated with 10^4.4^ TCID_50_ of PCV2 for 2 h and discarded the supernatant, Cepharaanthine and Curcumin were added and incubated for 48 h. Cells were treated with ROS assay kit and the changes of ROS were detected by Flow cytometer.

### Statistical analysis

CC_50_ was calculated using nonlinear regression and the results of “log (inhibitor) vs. response-variable slope” were analyzed using GraphPad Prism. The gray intensity of protein bolts was analyzed by Image J (National Institutes of Health, USA). Data generated were analyzed using “Bonferroni: Compare all pairs of columns” in the GraphPad Prism™ 5.0 software for one-way ANOVA implemented (GraphPad Software, Inc. California, USA), and were expressed as the mean ± standard error of the mean (SEM) of at least 3 repeated experiments. Different letters (a, b, c, d, etc.) indicate statistically significant difference to other groups (*p*<0.05).

## Supplementary information

**Additional file 1.** Original microscopic images of Cytotoxicity of 13 compounds on PK-15 cells.

**Additional file 2.** Original blot images of Fig. 2a, Fig. 5a and e. (a-f) Original blot images of Cap and GAPDH in the Fig. 2a, respectively. (g-j) Original blot images of cleaved caspase-3, Bcl-2, Bax, and GAPDH in the Fig. 5a, respectively. (k-n) Original blot images of cleaved caspase-3, Bcl-2, Bax, and GAPDH in the Fig. 5e, respectively.

## Data Availability

The datasets used and analysed during the current study are available from the corresponding author on reasonable request.

## Abbreviations

PCV2: Porcine circovirus type 2
PK-15: PCV-free pig kidney endothelial cell line
DMEM: Dulbecco’s modified eagle’s medium
MTT: 3-(4,5-dimethyithiazol-2-yl)-2,5-diphenyltetrazolium bromide
CPE: Cytopathologic effect
CR: Cytopathic ratio
MNTC: Maximum no-cytotoxic concentration
MSC: The maximum solubility concentration
CC_50_: 50% cytotoxic concentration
TCID_50_: 50% tissue culture infective dose
qPCR: Real time quantitative PCR
ROS: Reactive oxygen species
MMP: Mitochondrial membrane potential
