# Supplementary material for: Cepharanthine and Curcumin inhibited mitochondrial apoptosis induced by PCV2
Source: BMC Vet Res. 2020 Sep 18;16:345. doi: 10.1186/s12917-020-02568-0 (PMC7499946; doi:10.1186/s12917-020-02568-0)

**Supplem.** **Figure Captions**

**Cytotoxicity of 13 compounds on PK-15 cells detected by MTT.**

8 of gradient dilutions of each compound was prepared by 2-fold serial dilution and incubated with cells for 60 h. Microscopic images of the normal cell group, the cytopathic group and the safe concentration group were presented.

**Supplem. Fig**


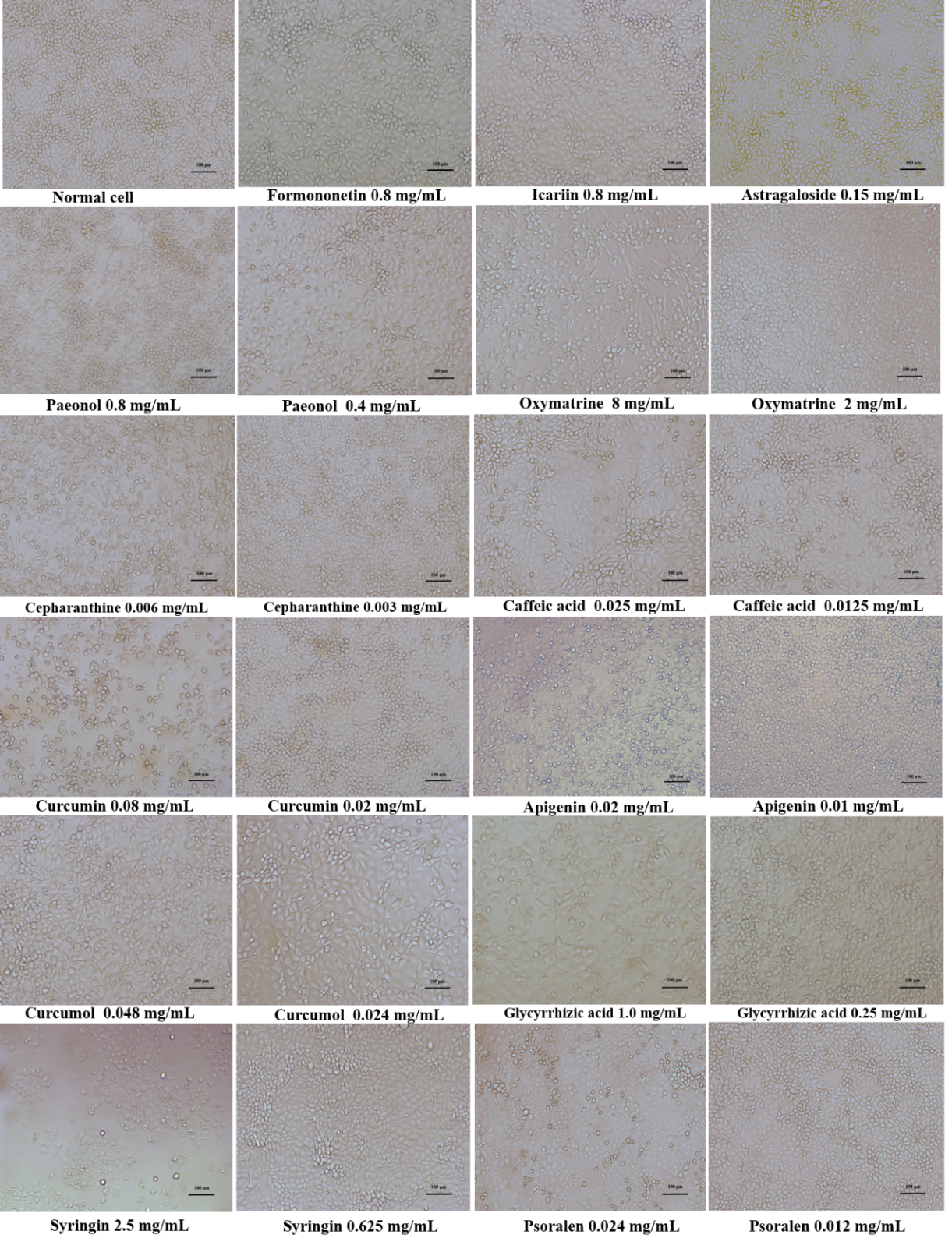

Supplement: Supplementary file 1 — Additional file 1. Original microscopic images of Cytotoxicity of 13 compounds on PK-15 cells. [file 12917_2020_2568_MOESM1_ESM.docx]
