# Supplementary material for: Cepharanthine and Curcumin inhibited mitochondrial apoptosis induced by PCV2
Source: BMC Vet Res. 2020 Sep 18;16:345. doi: 10.1186/s12917-020-02568-0 (PMC7499946; doi:10.1186/s12917-020-02568-0)
Supplement: Supplementary file 2 — Additional file 2. Original blot images of Fig. 2a, Fig. 5a and e. (a-f) Original blot images of Cap and GAPDH in the Fig. 2a, respectively. (g-j) Original blot images of cleaved caspase-3, Bcl-2, Bax, and GAPDH in the Fig. 5a, respectively. (k-n) Original blot images of cleaved caspase-3, Bcl-2, Bax, and GAPDH in the Fig. 5e, respectively. [file 12917_2020_2568_MOESM2_ESM.docx]

**Supplem. Figure Legends**

**Anti-PCV2 activities of Paeonol, Cepharanthine or Curcumin detected by Western blot and the mechanism of inhibition of PCV2-induced mitochondrial apoptosis by Cepharanthine or Curcumin.** Cells were incubated with 10^4.4^ TCID_50_ of PCV2 for 2 h, then tested compounds were added respectively and cultured for 48 h. Cells were collected and relative assay was performed. The expression of the Cap, cleaved caspase-3, Bcl-2 and Bax proteins were detected by Western blot. (a-f) Original blot images of Cap and GAPDH in the Fig. 3A after treatment with Paeonol, Cepharanthine and Curcumin, respectively. (g-n) Original blot images of cleaved caspase-3, Bcl-2, Bax, and GAPDH in the Fig. 6A after treatment with Cepharanthine and Curcumin, respectively.

**Supplem. Fig**


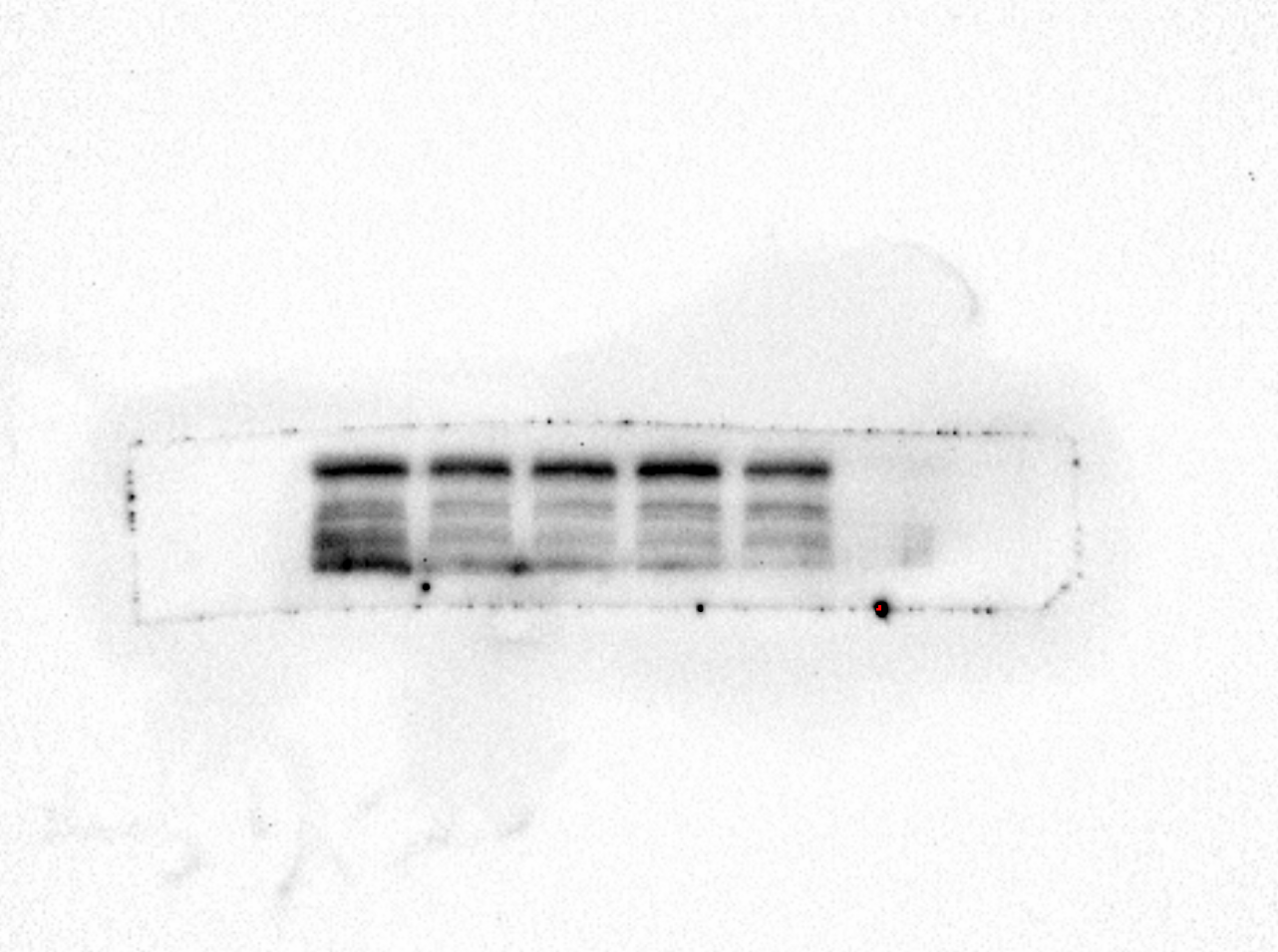


**Virus**

**Cell**

**Paeonol 0.2 mg/mL**

**Paeonol 0.1 mg/mL**

**Ribavirin 0.5 mg/mL**

**Paeonol 0.4 mg/mL**

**Cap**

**a Paeonol (Cap)**


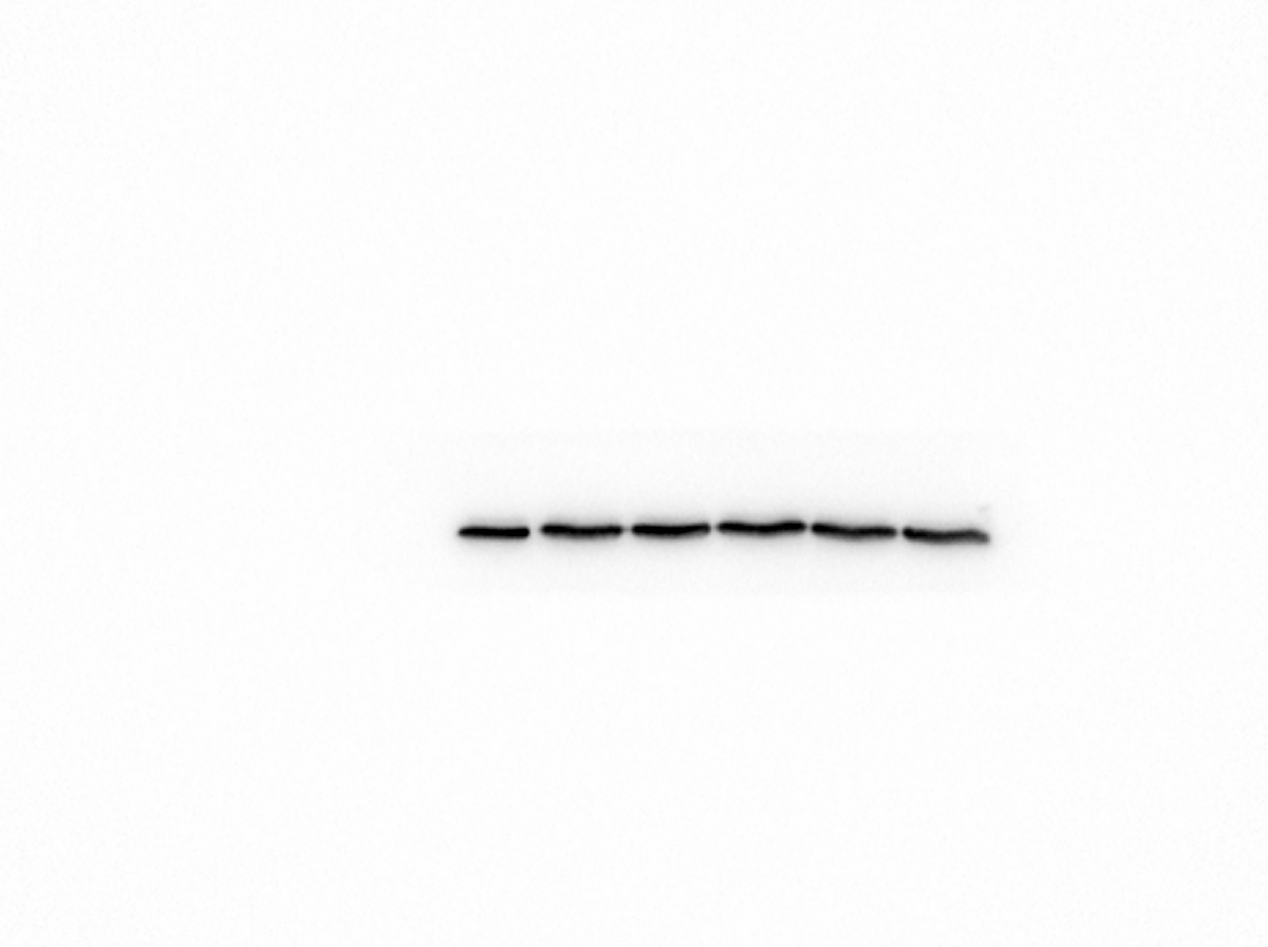


**Ribavirin 0.5 mg/mL**

**Virus**

**Cell**

**Paeonol 0.2 mg/mL**

**Paeonol 0.1 mg/mL**

**Paeonol 0.4 mg/mL**

**GAPDH**

**b Paeonol (GAPDH)**


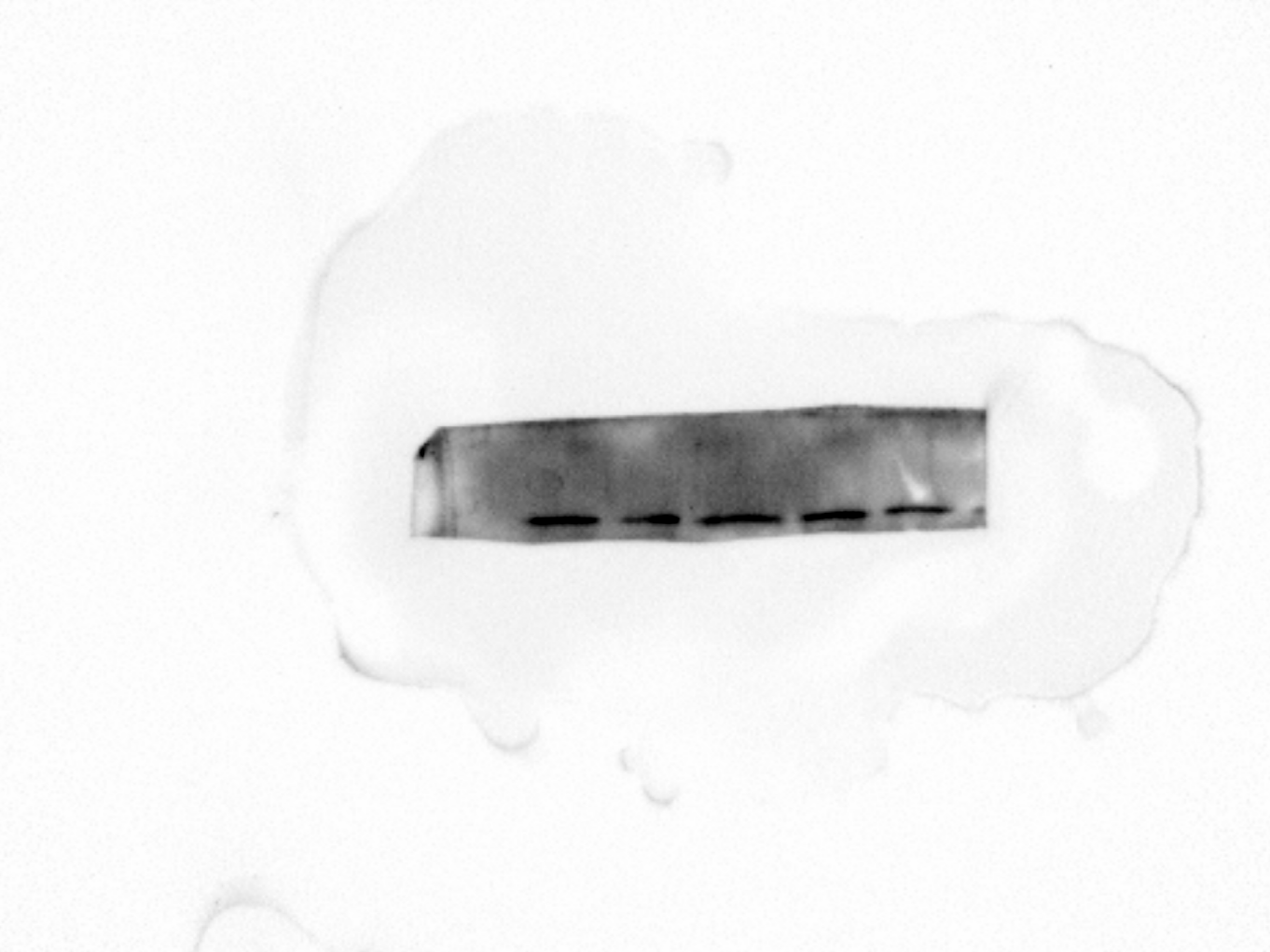


**Virus**

**Cell**

**Cepharanthine0.0015 mg/mL**

**Cepharanthine0.00075 mg/mL**

**Ribavirin 0.5 mg/mL**

**mg/mL**

**Cepharanthine0.003 mg/mL**

**Cap**

**c Cepharanthine (Cap)**


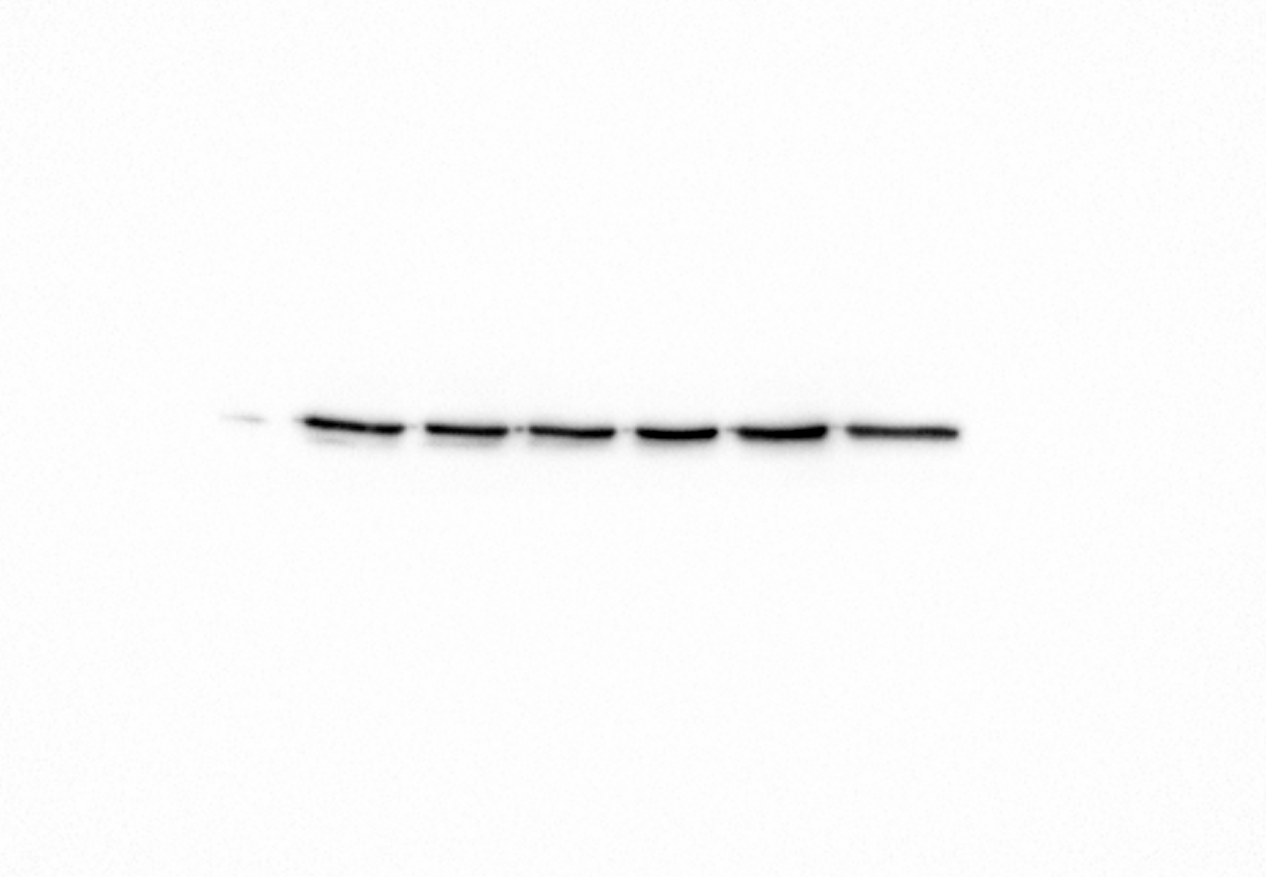


**Virus**

**Cell**

**Cepharanthine 0.0015 mg/mL**

**Cepharanthine 0.00075 mg/mL**

**Ribavirin 0.5 mg/mL**

**mg/mL**

**Cepharanthine 0.003 mg/mL**

**GAPDH**

**d Cepharanthine (GAPDH)**


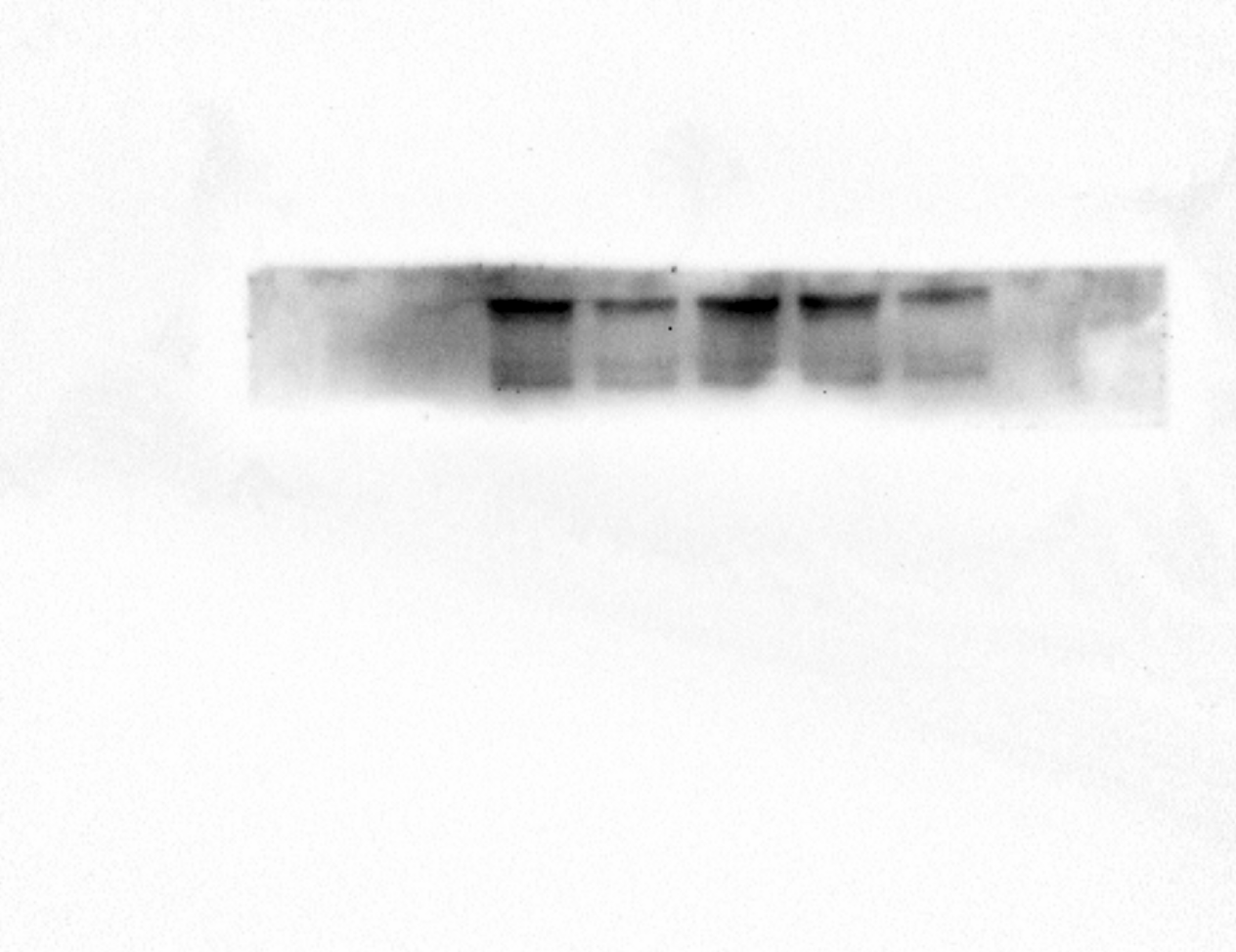

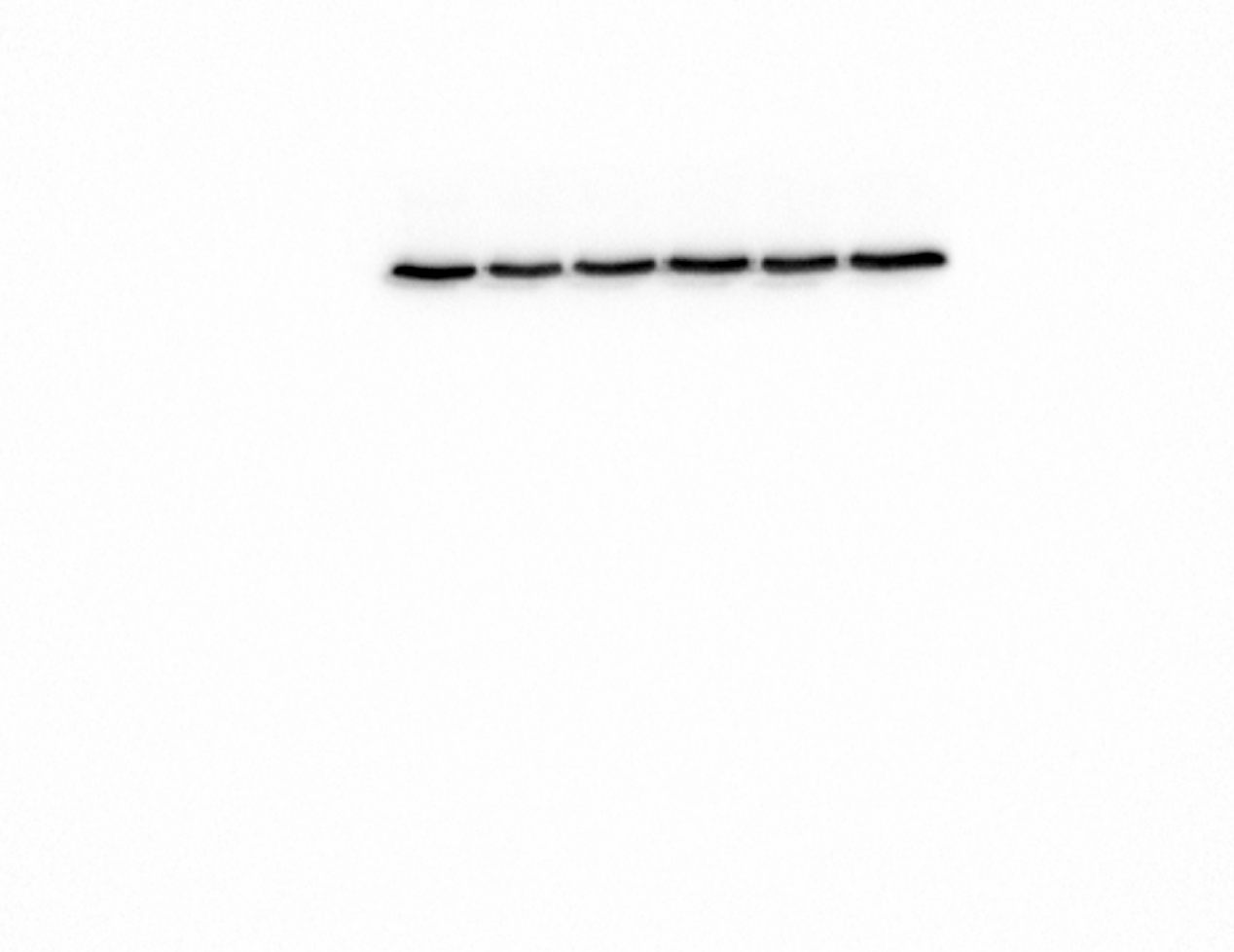


**e Curcumin (Cap)**

**f Curcumin (GAPDH)**

**Virus**

**Cell**

**Curcumin 0.01 mg/mL**

**Curcumin 0.005 mg/mL**

**Ribavirin 0.5 mg/mL**

**mg/mL**

**Curcumin 0.02 mg/mL**

**Cap**

**Virus**

**Cell**

**Curcumin 0.01 mg/mL**

**Curcumin 0.005 mg/mL**

**Ribavirin 0.5 mg/mL**

**mg/mL**

**Curcumin 0.02 mg/mL**

**GAPDH**


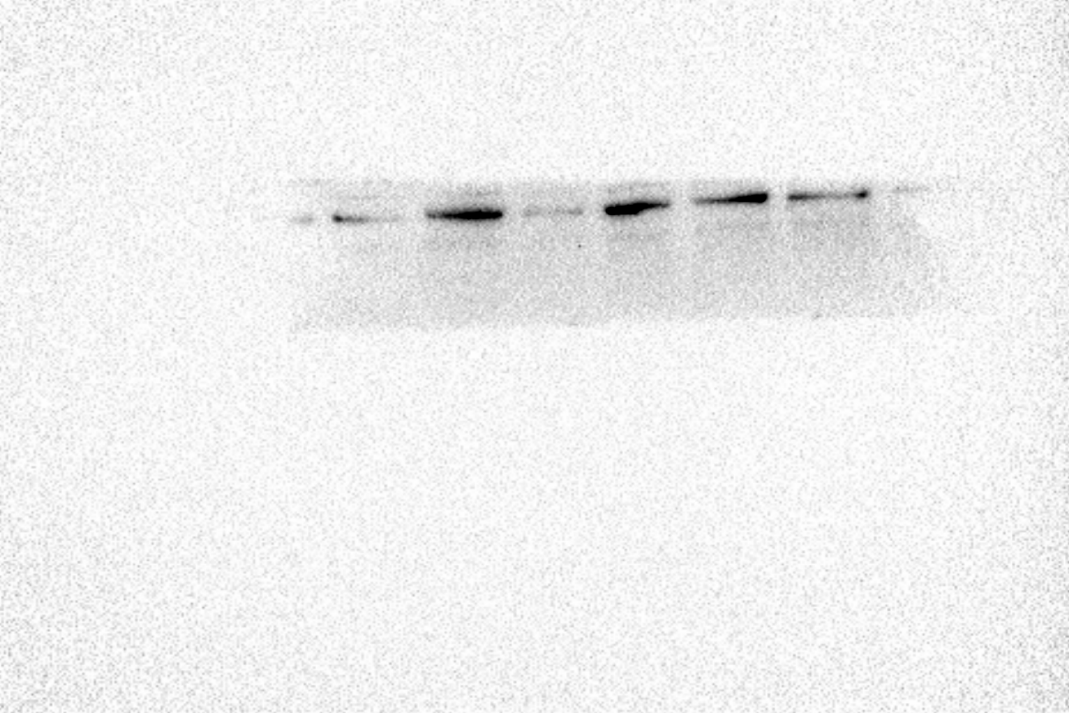

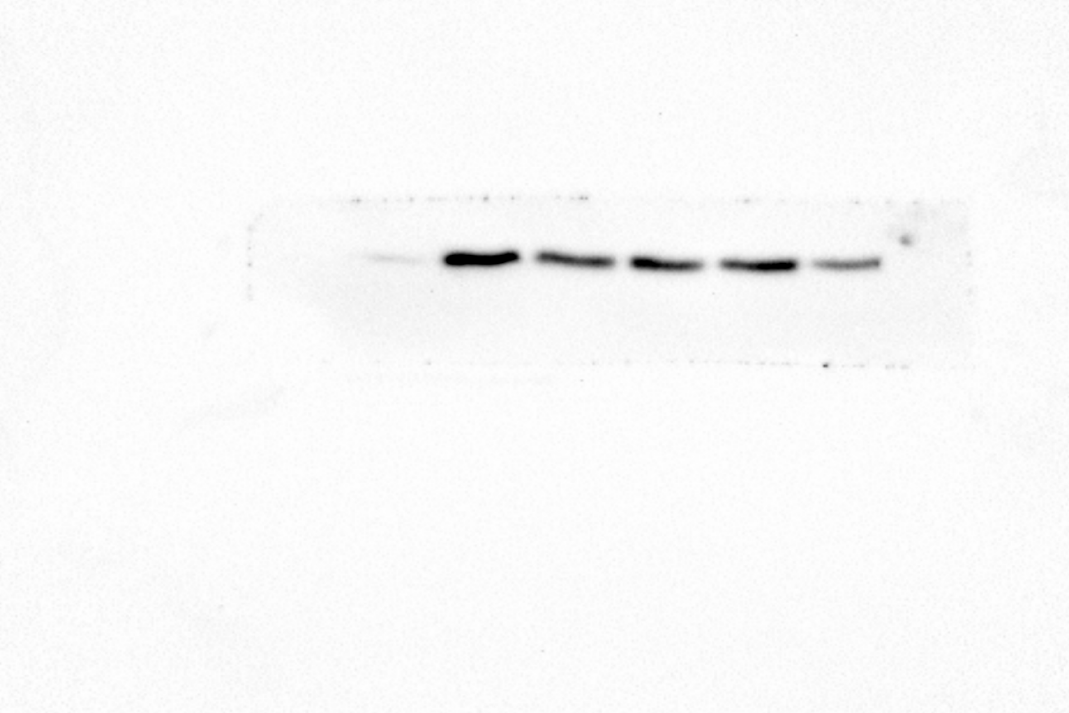


**Virus**

**Cell**

**Cepharanthine0.0015 mg/mL**

**Cepharanthine0.00075 mg/mL**

**Ribavirin 0.5 mg/mL**

**mg/mL**

**Cepharanthine0.003 mg/mL**

**Cleaved caspase-3**

**Virus**

**Cell**

**Cepharanthine0.0015 mg/mL**

**Cepharanthine0.00075 mg/mL**

**Ribavirin 0.5 mg/mL**

**mg/mL**

**Cepharanthine0.003 mg/mL**

**Bax**

**g Cepharanthine (cleaved caspase-3)**

**h Cepharanthine (Bax)**


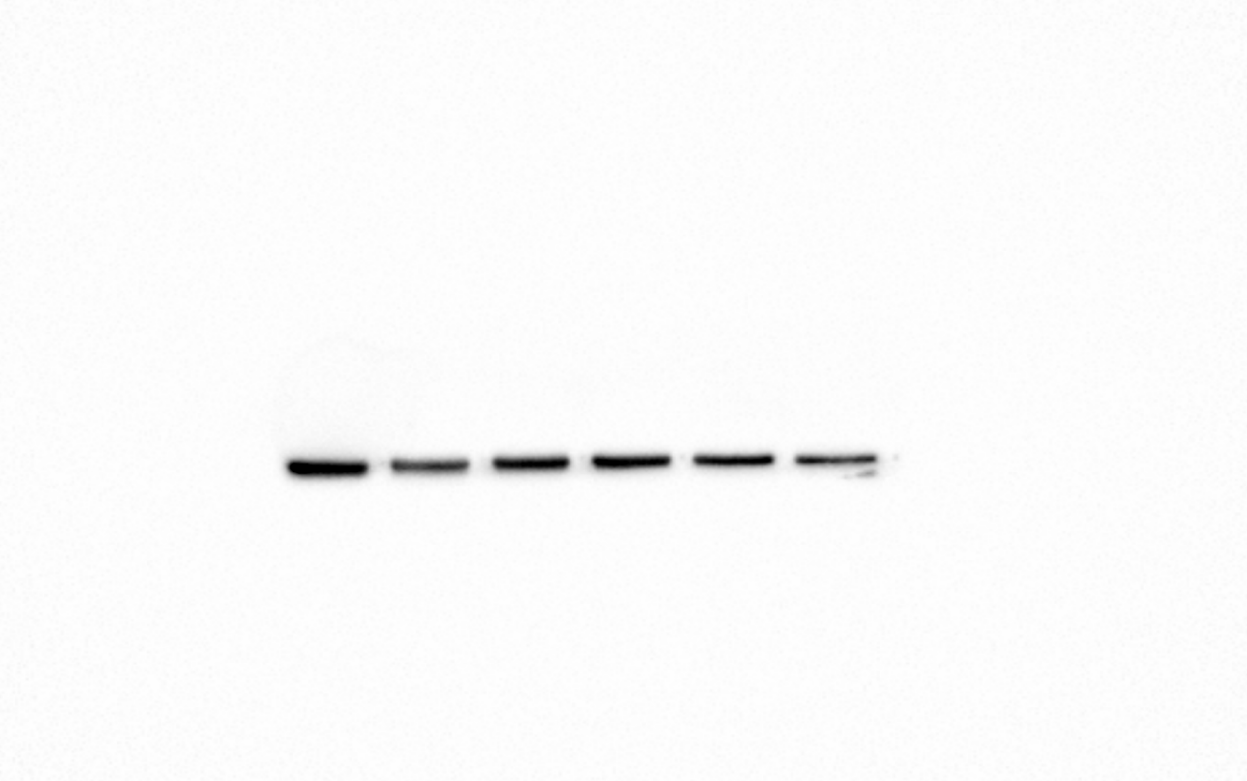


**Virus**

**Cell**

**Cepharanthine0.0015 mg/mL**

**Cepharanthine0.00075 mg/mL**

**Ribavirin 0.5 mg/mL**

**mg/mL**

**Cepharanthine0.003 mg/mL**

**Bcl-2**

**i Cepharanthine (Bcl-2)**


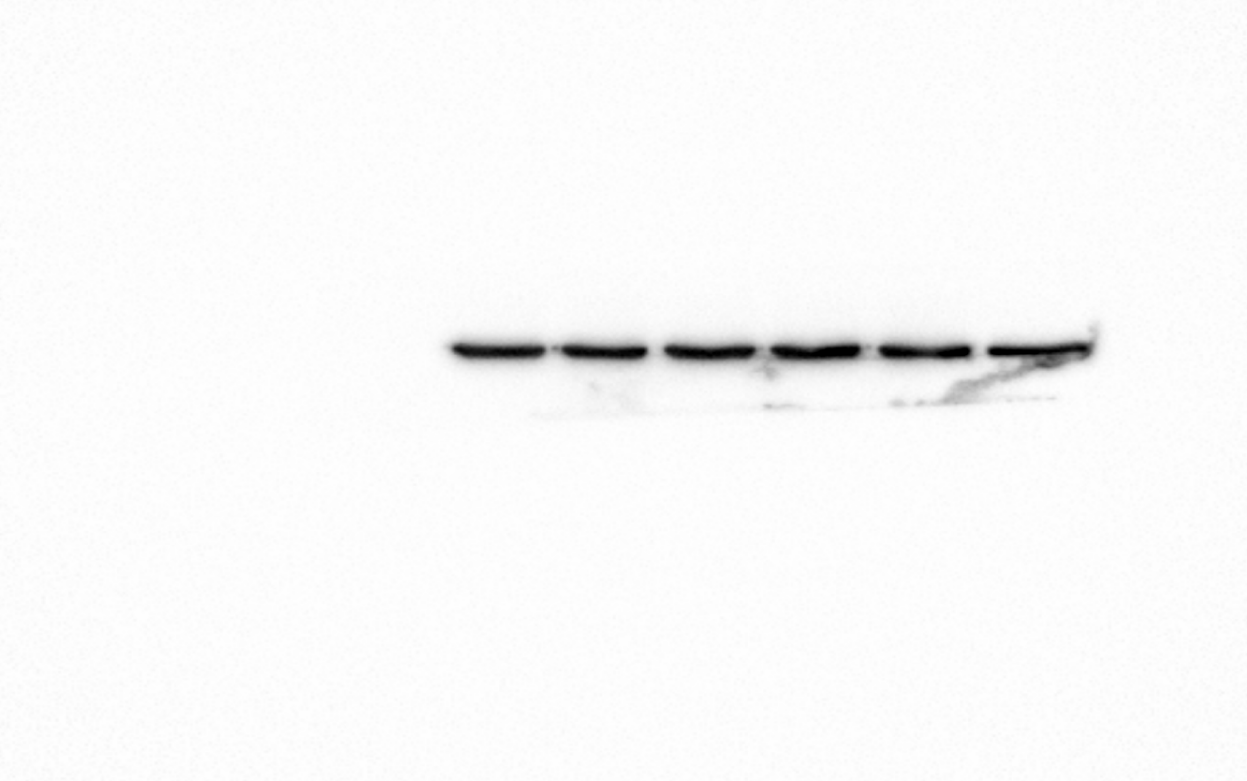


**j Cepharanthine (GAPDH)**

**Virus**

**Cell**

**Cepharanthine0.0015 mg/mL**

**Cepharanthine0.00075 mg/mL**

**Ribavirin 0.5 mg/mL**

**mg/mL**

**Cepharanthine0.003 mg/mL**

**GAPDH**


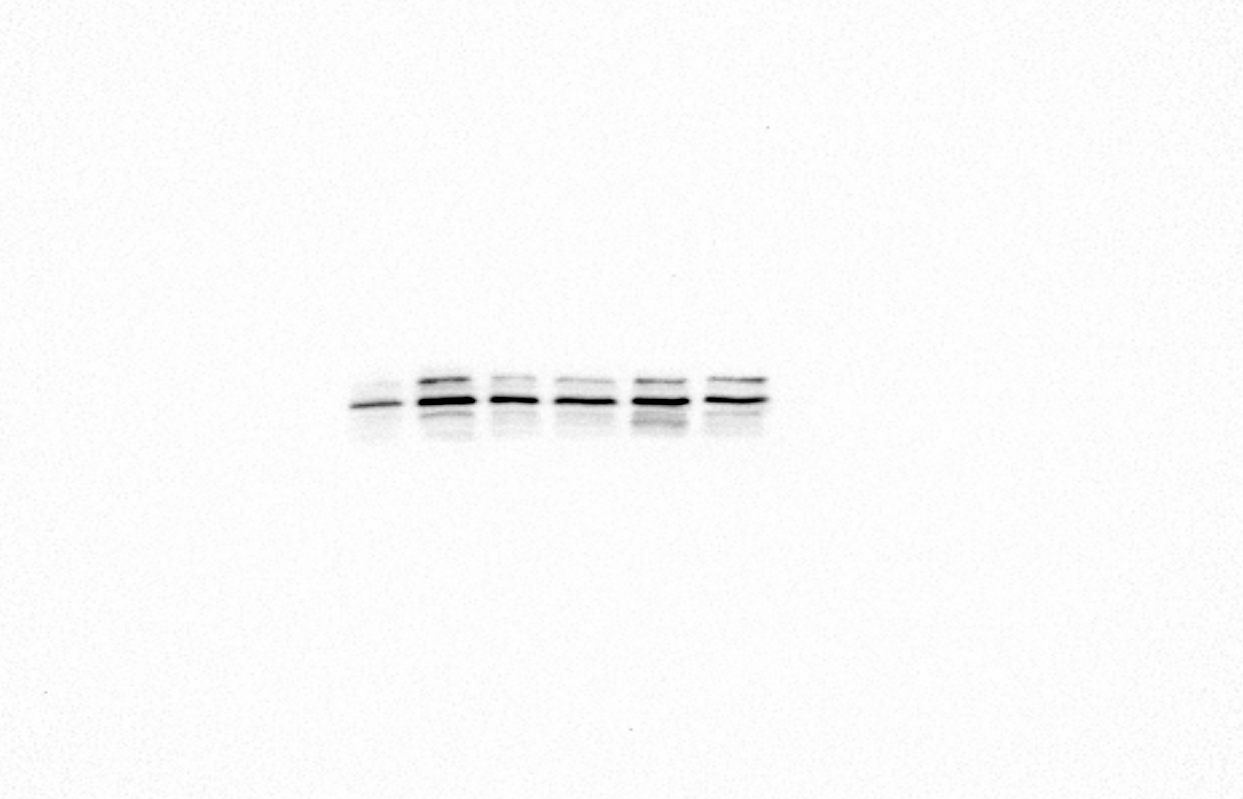


**Virus**

**Cell**

**Curcumin 0.01 mg/mL**

**Curcumin 0.005 mg/mL**

**Ribavirin 0.5 mg/mL mg/mL**

**Curcumin 0.02 mg/mL**

**Cleaved caspase-3**

**k Curcumin (cleaved caspase-3)**


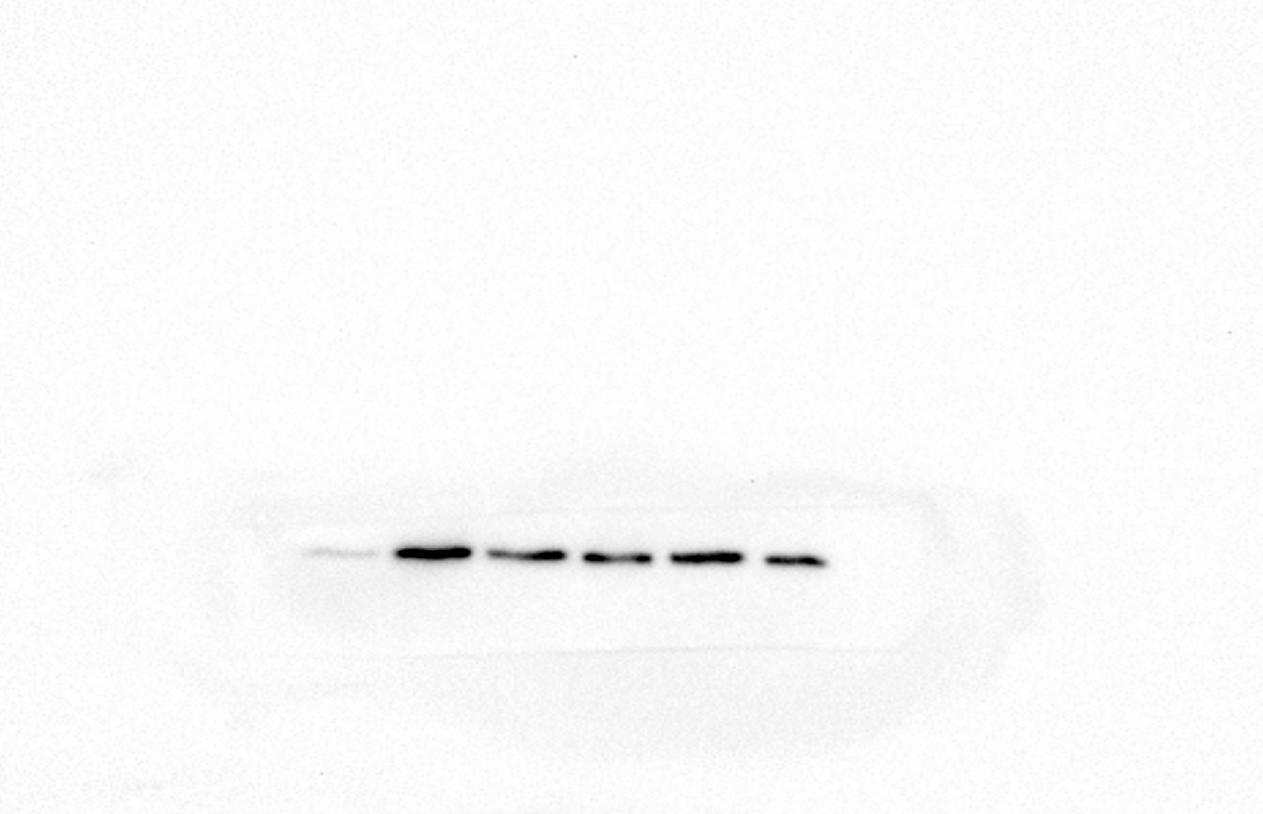


**Virus**

**Cell**

**Curcumin 0.01 mg/mL**

**Curcumin 0.005 mg/mL**

**Ribavirin 0.5 mg/mL mg/mL**

**Curcumin 0.02 mg/mL**

**Bax**

**l Curcumin (Bax)**


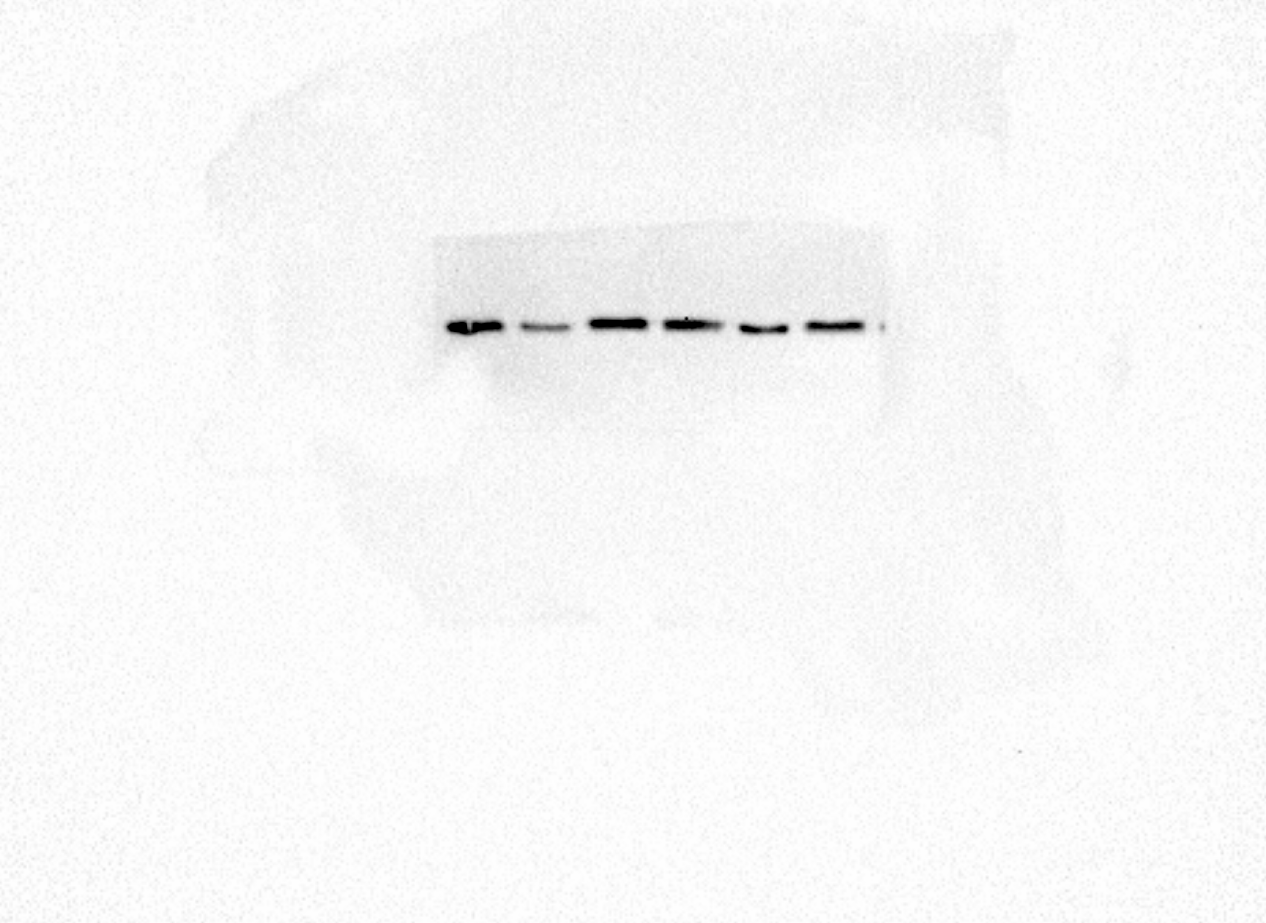


**m Curcumin (cleaved caspase-3)**

**Virus**

**Cell**

**Curcumin 0.01 mg/mL**

**Curcumin 0.005 mg/mL**

**Ribavirin 0.5 mg/mL mg/mL**

**Curcumin 0.02 mg/mL**

**Bcl-2**


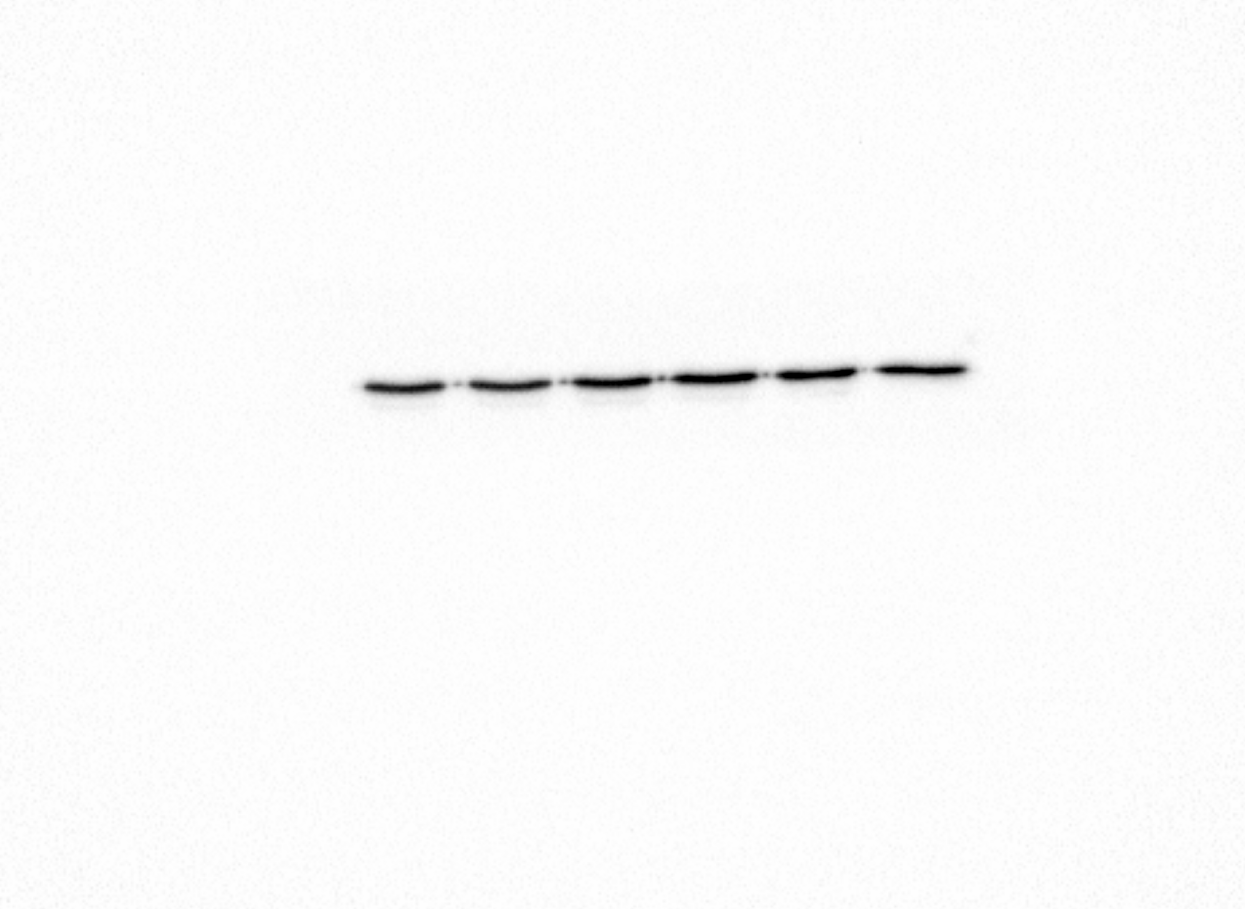


**Virus**

**Cell**

**Curcumin 0.01 mg/mL**

**Curcumin 0.005 mg/mL**

**Ribavirin 0.5 mg/mL mg/mL**

**Curcumin 0.02 mg/mL**

**GAPDH**

**n Curcumin (GAPDH)**
